# Evaluating the perceived impact and legacy of master’s degree level research in the allied health professions: a UK-wide cross-sectional survey

**DOI:** 10.1186/s12909-024-05582-0

**Published:** 2024-07-12

**Authors:** Terry Cordrey, Amanda Thomas, Elizabeth King, Owen Gustafson

**Affiliations:** 1 Oxford Allied Health Professions Research and Innovation Unit, Oxford University Hospitals NHS Foundation Trust, Oxford, OX3 9DU UK; 2 Centre for Movement, Occupational, and Rehabilitation Sciences, Oxford Brookes University, Oxford, OX3 0BP UK; 3 Barts Health NHS Trust, Royal London Hospital, London, E1 1BB UK

**Keywords:** Master’s degree, Research training, Allied health professions, Survey, Research capacity, Research impact

## Abstract

**Background:**

Post graduate master’s degree qualifications are increasingly required to advance allied health profession careers in education, clinical practice, leadership, and research. Successful awards are dependent on completion of a research dissertation project. Despite the high volume of experience gained and research undertaken at this level, the benefits and impact are not well understood. Our study aimed to evaluate the perceived impact and legacy of master’s degree training and research on allied health profession practice and research activity.

**Methods:**

A cross-sectional online survey design was used to collect data from allied health professionals working in the United Kingdom who had completed a postgraduate master’s degree. Participants were recruited voluntarily using social media and clinical interest group advertisement. Data was collected between October and December 2022 and was analysed using descriptive statistics and narrative content analysis. Informed consent was gained, and the study was approved by the university research ethics committee.

**Results:**

Eighty-four responses were received from nine allied health professions with paramedics and physiotherapists forming the majority (57%) of respondents. Primary motivation for completion of the master’s degree was for clinical career progression (*n* = 44, 52.4%) and formation of the research dissertation question was predominantly sourced from individual ideas (*n* = 58, 69%). Formal research output was low with 27.4% (*n* = 23) of projects published in peer reviewed journal and a third of projects reporting no output or dissemination at all. Perceived impact was rated highest in individual learning outcomes, such as improving confidence and capability in clinical practice and research skills. Ongoing research engagement and activity was high with over two thirds (*n* = 57, 67.9%) involved in formal research projects.

**Conclusion:**

The focus of master's degree level research was largely self-generated with the highest perceived impact on individual outcomes rather than broader clinical service and organisation influence. Formal output from master’s research was low, but ongoing research engagement and activity was high suggesting master’s degree training is an under-recognised source for AHP research capacity building. Future research should investigate the potential benefits of better coordinated and prioritised research at master’s degree level on professional and organisational impact.

**Supplementary Information:**

The online version contains supplementary material available at 10.1186/s12909-024-05582-0.

## Background

Higher levels of research engagement by healthcare organisations and clinicians are associated with improved organisational performance and clinical outcomes [1–3]. The Allied Health Professions (AHPs) comprise one third of the health and social care workforce in the United Kingdom and when engaged in research, offer substantial benefit to population health and organisational performance [4]. The strategic focus on AHP research has grown substantially in recent years. This includes the first ever national research and innovation strategy for AHPs in England, as well as clear strategic intention through AHP clinical research networks hosted by the National Institute for Health Research [5, 6]. These strategies reinforce the need for capacity building, engagement, and cultural improvements for advancing AHP research. Realising these ambitions has, to date, been limited by insufficient funding, career infrastructure, and organisational support [7].

Alongside the strategic ambitions for AHP research, is the increasing requirement for post-graduate master’s degree qualifications for career progression in academic, leadership and clinically advanced AHP roles. For example, 69% of Advanced Clinical Practitioners (ACP’s) state the requirement for master’s degree qualification for their current ACP role [8].

With few exceptions, a master’s degree award is dependent on the successful completion of a supervised research dissertation project. This is usually accompanied by taught research methodology to support the development of research knowledge and skills. Master’s degree research ideas are conceived in a variety of ways, either as stand-alone projects, supplied by a university academic as one part of a larger programme of work, or developed in collaboration with a health service [9]. AHP research projects developed collaboratively between health and academic centres are more likely to be widely disseminated, impactful on clinical practice, and lead to further research compared to projects undertaken exclusively within a university setting [10].

Despite the high cumulation of training and research at this level over the years, the broader impact on clinical services, employing organisations, and the wider research community is currently unknown [11]. Beyond the fulfilment of individual learning objectives, it is difficult to determine what real-world impact AHP master’s research offers in terms of original knowledge contribution. Similarly, the rate of conversion of AHP master’s degree research to peer reviewed publications or conference proceedings remains unexplored [12]. This situation risks a low return on investment in terms of the generation and translation of knowledge to address the challenges faced by AHPs in healthcare practice [13]. Responsible practice in AHP post graduate training and research should, in part, be concerned with reducing waste that arises from decisions about what research to prioritise, as well as educational benefit to the individuals [14]. Aligning and coordinating more AHP master's degree research activity through collaboration may prevent AHP dissertations entering the “relevance waste quadrant” [15]. Models of portfolio research, which are coordinated efforts to address the highest priority knowledge gaps through research collaborations, represent an alternative approach to the current system [16].

## Aims

The primary aim of our study is to evaluate the perceived impact and sustained effect of master’s degree research dissertation projects on AHP research capacity, capability, and clinical practice. In doing this, we have set out five supporting objectives:

- To understand how master’s degree research dissertation questions were determined.
- To establish the rate of conversion of master’s degree research to traditional measures of research output and dissemination.
- To establish whether successful completion of master’s degree research promotes the maturation of ongoing research active clinicians.
- To determine the perceived impact of research skills developed through master’s degree completion on AHP research capacity building within individuals and organisations.
- To determine the perceived impact of master’s degree research on clinical practice and services.

## Methods

An online cross-sectional survey design was chosen as the method to conduct this study, and it is being reported according to the consensus-based checklist for reporting of survey studies [17]. A bespoke survey was constructed using Microsoft Forms software and was hosted online via Microsoft Office 365. The survey comprised 27 questions arranged into sections to collect data on participant demographics, and the experience, outcomes, and perceived impact of master’s degree training and completion of a research dissertation project (see additional file 2 in supplementary information). To develop the survey, a pilot survey was undertaken using four qualified AHP volunteers to appraise the structure, content, and readability of the questions. Feedback from the pilot was used to revise and finalise the survey.

The target population were AHPs, which is an umbrella term for fourteen different professions usually employed in a variety of roles across health, care, academic, and voluntary sectors (https://www.england.nhs.uk/ahp/role/). Participants were eligible to take part if they were 1) qualified AHPs currently working in the United Kingdom (UK), 2) held a post graduate master’s degree award, and 3) were able to provide informed consent. Participants were ineligible if their master’s degree was obtained as a pre-registration qualification, and they did not meet the other inclusion criteria. A target sample size of 139 was calculated by estimating the proportion of all registered AHPs in the UK holding a master’s degree qualification. This estimation was determined by profiling the qualifications of AHP staff in two large National Health Service (NHS) teaching hospitals. To account for a sampling calculation error, a confidence interval (95%) and margin of error (5%) threshold were applied accordingly (see additional file 3 in supplementary information).

Participant recruitment was achieved through advertising on social media platforms, and via newsletters and bulletins circulated by AHP professional and clinical interest groups. Participant information was provided outlining the study details, anonymity of survey responses, and the requirement to provide informed consent and eligibility at the start of the online survey. Those taking part were asked to reflect on their experiences of completing a post graduate master’s degree and research dissertation project in relation to its impact and legacy. The ‘one response per participant’ feature was enabled to prevented multiple completion of the survey by the same participant. The survey was live for data collection for three months running from October to the end of December 2022. During data collection, several efforts were made to promote the survey through social media to increase participation.

The survey data was analysed in two ways. First, descriptive statistics were used to analyse numerical, multiple choice, and ordinal scale data. Second, free text responses providing reflective accounts and experiences underwent coding and content analysis using NVivo software (version 12).

This study was approved by the university research ethics committee (registration number: 221613) and was conducted in accordance with the principles of good clinical practice.

## Results

The survey received 84 responses from nine of the fourteen allied health professions, which represents 60% of the target sample of 139. The majority of responses were from physiotherapists (*n* = 40, 47.6%) and respondents had been qualified for a median (IQR) of 18 years (12–23). Respondents worked in a variety of clinical specialties, with emergency/pre-hospital medicine (*n* = 18, 21.4%), neurology (*n* = 12, 14.3%) and critical care (*n* = 11, 13.1%) the most common. Most respondents had completed their master’s degree after 2010 (*n* = 68, 81%) and were employed at band 6 grade when starting (*n* = 39, 46.4%). Most respondents worked in the NHS (*n* = 78, 92.3%) and had undertaken a Master of Science (MSc) award (*n* = 70, 83.3%). Most participants were employed in a higher paid position after completing their master's degree (*n* = 62, 73.8%). The full characteristics of the respondents are detailed in Table 1.

**Table 1:** Survey respondent characteristics

	**n (%)**
Profession	
Chiropodist/podiatrist	1 (1.2)
Dietitian	4 (4.8)
Occupational Therapist	7 (8.3)
Operating Department Practitioner	1 (1.2)
Orthoptist	3 (3.6)
Paramedic	18 (21.4)
Physiotherapist	40 (47.6)
Radiographer	2 (2.4)
Speech and Language Therapist	8 (9.5)
Starting band	
5	5 (6)
6	39 (46.4)
7	31 (36.9)
8a	6 (7.1)
8b	1 (1.2)
Other	2 (2.4)
Current band	
6	11 (13.1)
7	29 (3.5)
8a	24 (28.6)
8b	16 (19)
8c	1 (1.2)
Other	3 (3.6)
Years qualified (median, IQR)	18 (12–23)
Clinical Specialty	**n (%)**
Paediatrics	5 (6)
Neurology	12 (14.3)
Critical care	11 (13.1)
Oncology	3 (3.6)
Cardiorespiratory	9 (10.7)
Learning disabilities	1 (1.2)
Musculoskeletal	7 (8.3)
Orthoptics	3 (3.6)
Voice/upper airways	2 (2.4)
Theatres	1 (1.2)
Adult rehabilitation	2 (2.4)
Social Care	2 (2.4)
Emergency/pre-hospital medicine	18 (21.4)
Artificial Intelligence	1 (1.2)
Education	1 (1.2)
Nutrition	3 (3.6)
Advanced practice	1 (1.2)
Mental health	1 (1.2)
Primary reason for master’s study	
Career progression (clinical)	44 (52.4)
Career progression (leadership)	3 (3.6)
Career progression (research)	20 (23.8)
Career progression (education)	4 (4.8)
Personal interest	9 (10.7)
Employer expectation	2 (2.4)
Others	2 (2.4)
Type of master’s degree competed	
MA	2 (2.4)
MSc	70 (83.3)
MRes	11 (13.1)
Other	1 (1.2)

Respondents predominantly formed their dissertation research questions from their own area of interest (Table 2). Less than 10% of the dissertation questions were based on published research priorities or set by the Higher Education Institute (HEI), regional or local healthcare organisation/collaborative (*n* = 7, 8.3%). A variety of methodologies were used to conduct the master’s research dissertation with evidence synthesis being the most common (*n* = 30, 35.7%).

**Table 2:** Dissertation details

	**n (%)**
Formation of master’s dissertation question	
From own ideas	58 (69)
Based on published research priorities	4 (4.8)
Discussion with supervisor	16 (19)
Pre-set by university	1 (1.2)
Pre-set by university and healthcare organisation collaboration	1 (1.2)
Pre-set by regional or national research collaborative	1 (1.2)
Other	3 (3.6)
Research method of dissertation	
Evidence synthesis	30 (35.7)
Service evaluation	18 (21.4)
Qualitative (survey/questionnaire)	7 (8.3)
Qualitative (interview/focus group)	19 (22.6)
Action/participatory research	5 (6)
Quantitative (secondary analysis)	7 (8.3)
Quantitative (observational)	13 (15.5)
Quantitative (experimental)	18 (21.4)
Other	8 (9.5)
Output from research dissertation	
Local presentation	44 (52.4)
Conference poster	37 (44)
Conference presentation	31 (36.9)
Published abstract	30 (35.7)
Published in clinical interest group journal/newsletter	12 (14.3)
Published in peer reviewed journal	23 (27.4)
Published in blog	2 (2.4)
Published in profession specific circulation	9 (10.7)

Formal research output from the dissertations was low (Table 2). Half the dissertations were presented at a local research symposium (*n* = 44, 52.4%), 27.4% (*n* = 23) were published in a peer reviewed journal, and over a third of dissertations had no output at all (*n* = 30, 35.7%). Master’s degree programmes contributing to the peer reviewed publications as a proportion of students were Master by Research (MRes) (*n* = 5, 45.5%), and MSc (*n* = 18, 25.7%).

Of the dissertations formed through the individual's own ideas, 27.6% (*n* = 16) were published in a peer reviewed journal, compared to 57.1% (*n* = 4) of those set through research priorities, or the HEI/healthcare organisation. The most common methodologies published in a peer review journal were evidence synthesis (*n* = 7, 30.4%), qualitative interviews/focus groups (*n* = 6, 26.1%) and quantitative experimental studies (*n* = 6, 26.1%). The methodology of dissertation projects with the highest proportion of peer reviewed journal publication was qualitative interviews/focus groups (*n* = 7, 36.8%).

**Fig. 1:**
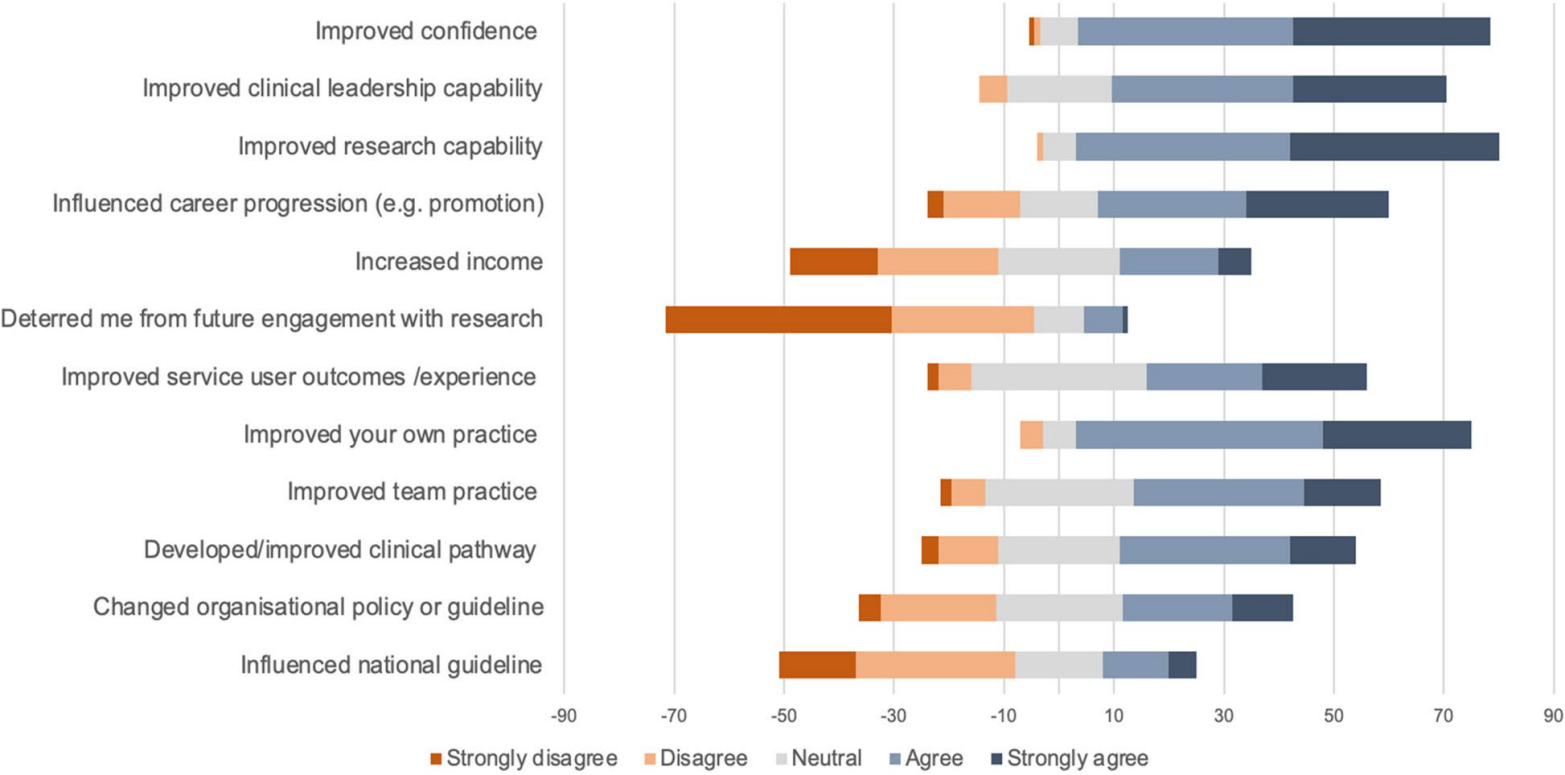
Perceived impact of master’s degree research on professional and clinical service development

The respondents reported their master's degree dissertation as having a positive impact on their professional development (Fig. 1). Qualitative content analysis of the free text responses demonstrated that respondents felt the dissertation increased their research capability and confidence at multiple stages of the research process while providing opportunities for networking and collaborations.

Most participants continued to engage in research activities after their dissertation (*n* = 65, 77.4%) through supporting others (*n* = 63, 75%), taking part in formal research projects (*n* = 57, 67.9%) and publishing research papers (*n* = 41, 48.8%) (Table 3). Less than ten percent (9.5%, *n* = 8) reported being deterred from undertaking further research (Fig. 1).

**Table 3:** Post-dissertation research activity

	**n (%)**
Embarked on a higher research degree (PhD)	31 (36.9)
Taken part in formal research projects	57 (67.9)
Received research grants/funding	32 (38.1)
Published research papers	41 (48.8)
Protected research time	26 (31)
Work full time in research/academia	11 (13.1)
Help others to develop research skills and undertake projects	63 (75)
Not engage in research activity	19 (22.6)

The wider perceived impact of the dissertation on services in which the respondents worked was more varied (Fig. 1). Improved service user outcome/experience and team practice was reported by 60.7% (*n* = 51) and 53.6% (*n* = 45) respectively. Analysis of free text responses demonstrated wide ranging perceived impact on services from no local impact to improved team education, service delivery and application of evidence-based practice.

## Discussion

Our study evaluated the perceived impact of master's degree level research on AHP professional development, research capacity, and clinical practice. Our findings indicate a relatively low level of dissemination and formal output arising from master’s degree research, but a high perception of impact on individual AHPs and the clinical services in which they work. The level of ongoing engagement in research activity following master’s degree completion was high indicating a positive legacy in this respect. The degree to which this meaningfully contributes to AHP research capacity building requires further investigation.

The majority (69%) of master’s degree research questions were developed from the respondent’s own ideas rather than drawing on published research priorities or collaborations between health and academic organisations. The limited use of research priorities may be explained by a potential lack of awareness. A qualitative study of 95 AHPs working in Australia found that in the absence of a recognised framework to guide research prioritisation, individual clinicians conducted research in areas important to them [18]. Pursuing individual preferences in this way stemmed from evaluations of their personal work, departmental policies or procedures, models of care innovation, and a clear preference for research which “tested solutions”. Similarly, Amalkumaran et al. (2016) explored critical care research priorities and found that research topics suggested by professional sub-groups tended to be related to their daily practice rather than broader research priorities [19].

It is also possible that the choice of research question is influenced by the career motivation of the individual AHP. A UK wide cross-sectional survey of AHPs working in health and social care reported primary motivators for research participation were to develop skills (80%) and increase job satisfaction (63%), rather than contribute to the prioritised evidential knowledge base [20]. Davis et al. (2019) also recognise this self-actualising motivation for research participation in their AHP cohort [18]. It is possible that the debut, non-commissioned research activity introduced by master’s level academic programmes emphasises *process* over *content*, decreasing the alignment of research activity with known research priorities.

We found a low conversion rate from master’s dissertation completion to formal research output. This is well illustrated in that just one in four (27.4%) master’s theses resulted in a peer-reviewed publication. Similar publication rates have been reported in master’s students of other healthcare disciplines; these are also considered low by way of expected research output [21]. Understanding this further is challenging due to the limited research in this subject area, which suggests a lack of interest and/or perceived importance. However, there are two key issues that arguably counter this view. First, master’s degree research projects are typically approved by a university research ethics committee, and thus are guided by the principle that the value in their conduct and knowledge contribution should outweigh the burden or risks to participants [22]. Fidelity to this principle can only be meaningfully appraised if the results are published for wider critical evaluation. Second, AHP skill and success level in research activities, such as writing for peer-reviewed publication is widely and consistently reported as low [23–25]. This clearly represents an area for improvement for AHPs and failing to challenge the development of this skill in those undertaking post graduate level research seems counter intuitive. Higher rates of master’s degree research publication could offer a meaningful contribution to AHP research capacity building, since our findings suggest there is continued engagement in research activity from this group beyond completion of their studies.

Respondents to our survey indicated a good level of research engagement after master’s degree training. Over three quarters reported continued involvement in research beyond the completion of their programme. This finding supports the idea that research education is a key lever and greatly needed to successfully build AHP research capacity [26, 27]. However, the degree to which master’s degree training translates to growth in the research capacity of individuals has not been subject to causal investigation. Proxy indicators of individual research capacity from our cohort can be found in the self-reported high levels of research confidence and capability derived from master’s degree training (Fig. 1) and ongoing research activity. This activity included 60% taking part in formal research projects, around half had published research papers, and over a third had embarked on a higher research degree. The lack of previous research in this area makes it challenging to fully contextualise our findings, but in conducting our study, we have set out a benchmark for the perceived impact of masters degree training on individual AHP research capacity for future investigation.

We explored higher level outcomes of master’s degree training on research capacity building, such as those that might influence policy, career pathways, and organisational practice. Using the Kirkpatrick-Barr model of educational outcomes, we found the activity and outcomes from our cohort aligned best to an individual learner level [28]. This finding is typical of outcomes from education at this level, which centre largely on the individuals through self-reported satisfaction and perceptions of learning [29]. Understanding the impact of research education and training in relation to higher Kirkpatrick-Barr outcomes requires objective and longitudinal evaluation of research metrics and impact at organisational and system level [30]. This is likely to include contributions to larger programmes of work requiring large grant awards, significant publications, and translation of those research findings to health organisation and system level innovation [31, 32]. Research capacity building at this level is known to be challenging due to the inherent complexities involving political, financial, structural, and cultural factors [33]. To overcome this, the use of theoretical frameworks has been suggested to help conceptualise and integrate a culture and proliferation of AHP research at various health system structural levels [34]. The positioning of AHP master’s degree training and research activity as part of this may foster greater academic-health system collaboration for professional, service user, and population benefit [35].

The perceived impact of master’s degree research included improvements to service user outcomes, clinical pathways, and organisational policies and/or guidelines. Research impact, defined as the demonstrable benefit of research to individuals and society, is complex and requires wide stakeholder engagement to determine whether research has addressed known priorities through effective translation of knowledge from its findings [36]. The self-generated research questions and low level of dissemination and output reported by our cohort suggests a degree of dissonance between the level of perceived impact versus what is measurably impactful to clinical services and end users. This difference may be explained by the challenges in defining and quantifying research impact for novice researchers, which is described as an ambiguous and subjective concept [37]. It is therefore not surprising to see the highest levels of reported impact from our cohort was on their own professional development in terms of improved confidence, leadership and research capability, and clinical practice development. Without a more objective assessment of the wider impact from the research undertaken at this level, it is difficult to reconcile its actual impact. The emergence of assessment frameworks, such as the visible impact of research tool, make it accessible for relatively inexperienced researchers to understand how their research has led to visible changes and impact on services and other research consumers [38].

### Strengths and limitations

A key strength of our study lies in its novelty; we believe it to be the first to evaluate the perceived impact of research undertaken by AHPs at master’s degree level. This represents an important first step in highlighting the conduct and contribution of research undertaken at this level, as well providing opportunities to improve future practice and impact. There are several limitations to our study. We only managed to recruit 60% of our target sample via a non-probability sampling technique, which included a lack of representation from five of the 14 professions. This means our findings are vulnerable to sampling bias by potentially excluding AHPs who do not use social media or subscribe to clinical interest groups, which were the two main platforms for our recruitment. Our recruitment practice and the method of a self-reporting survey means our findings are not generalisable to the wider AHP population and they should be interpreted with these limitations in mind. A further limitation is the disproportionate representation of two of the fourteen allied health professions. Responses from paramedics and physiotherapists constituted 57% of our data with very few responses from seven other professions and no responses from five of the professions.

## Conclusion

The perceived impact of AHP master’s degree training and research was highest on individual development rather than service and organisation outcomes. This is likely to derive from the individual motivation in undertaking post-graduate study and self-determined research dissertation focus. Whilst the formal research output arising from the master’s research was relatively low, the legacy in terms of ongoing research engagement and activity was positive indicating that master’s degree completion maybe an under-recognised source of AHP research capacity building. Our study provides novel insights into the perceived impact of AHP master’s degree level research. Future research should explore the feasibility and benefits of coordinating AHP master’s degree research with local or national priorities to understand the impact beyond that realised at an individual level.

### Supplementary Information

Supplementary Material 1.

Supplementary Material 2.

Supplementary Material 3.

## Data Availability

All data generated or analysed during this study are included in this published article [and its supplementary information files].

## Abbreviations

AHP: Allied Health Professions
ACP: Advanced Clinical Practitioner
UK: United Kingdom
NHS: National Health Service
NVivo: Qualitative data analysis software
IQR: Interquartile Range
MSc: Master of Science
HEI: Higher Education Institute
MRes: Masters by Research
MA: Master of Art
PhD: Doctor of Philosophy
