# Supplementary material for: Evaluating the perceived impact and legacy of master’s degree level research in the allied health professions: a UK-wide cross-sectional survey
Source: BMC Med Educ. 2024 Jul 12;24:750. doi: 10.1186/s12909-024-05582-0 (PMC11241887; doi:10.1186/s12909-024-05582-0)
Supplement: Supplementary file 2 — Supplementary Material 2. [file 12909_2024_5582_MOESM2_ESM.pdf]

Evaluating the impact and legacy of AHP master's degree level research projects.

Thank you for your interest in our study. We would very much appreciate around 15 minutes of your time to help us understand the impact that AHP master's degree research dissertation projects have on individuals, services and research activity.

Allied Health Professionals (AHPs) are increasingly required to gain master's degree qualifications to progress into academic, leadership and clinically advanced roles. A mandatory requirement for this award is the completion of a research dissertation module, which usually means conducting a research project approved by the university's research ethics committee. An estimated 10% of AHPs in the United Kingdom have a master's degree qualification, which in theory, translates, as a relatively large repository of research project experience and output. Despite this, there is little indication to suggest that this aggregated body of work and collective experience is better than the sum of its parts. The benefits appear to be largely borne by the individual in terms of future income change, role competency and job satisfaction. The broader impact and legacy of this work is not well understood in terms of its contribution to the research community, particularly AHP research capacity building and the quality and safety of clinical practice. The purpose of this study is to gain the perspective of AHPs who have completed a master's degree research dissertation project to understand the broader impact of their experience, skill acquisition and research findings. The findings from this study will help determine the scope and future opportunities to improve the coordination, collaboration and impact of research undertaken at this level.

You are being invited to take part in a research study. Before you decide whether or not to take part, it is important for you to understand why the research is being done and what it will involve. Please take time to read the following information carefully, available here https://docs.google.com/document/d/13qByxmw1P70AVB-UHyLlEg2VM8HNSsHa/edit?usp=sharing&ouid=114603453785517802888&rtpof=true&sd=true (https://docs.google.com/document/d/13qByxmw1P70AVB-UHyLlEg2VM8HNSsHa/edit?usp=sharing&ouid=114603453785517802888&rtpof=true&sd=true)

* Required

1. To take part in this survey, you must be a qualified Allied Health Professional and have completed a (post graduate) master's degree relevant to your professional field, or role. Please indicate whether you meet this criteria to take part. *

   There are 14 AHP professions, please take a look here if you are not sure which professions are included: https://www.england.nhs.uk/ahp/role/ (https://www.england.nhs.uk/ahp/role/)

   ◯ Yes

   ◯ No

2. Consent 1: I confirm that I have read and understand the participant information sheet for the above study and have had the opportunity to ask questions. *

   Please read the participant information sheet, which can be found here (insert hyperlink to PIS)

   ◯ Yes

   ◯ No

3. Consent 2: I understand that my participation is voluntary and that I am free to withdraw prior to submitting my response without giving a reason. *

   Because this is an anonymous survey, if you submit the survey, we will not be able to withdraw your data.

   ◯ Yes

   ◯ No

4. Consent 3: I agree that an anonymised data set, gathered for this study may be stored in a specialist data centre/repository relevant to this subject area for future research *

   ◯ Yes

   ◯ No

5. Consent 4: I agree to take part in this study. *

   ◯ Yes

   ◯ No

6. Please state your job title. *

7. Please select your profession. *

- ○ Art Therapist
- ○ Chiropodist/Podiatrist
- ○ Dietitian
- ○ Drama Therapist
- ○ Music Therapist
- ○ Occupational Therapist
- ○ Operating Department Practitioner
- ○ Orthoptist
- ○ Osteopath
- ○ Paramedic
- ○ Physiotherapist
- ○ Prosthetist/Orthotist
- ○ Radiographer
- ○ Speech & Language Therapist

8. Please select the grade of your role at the time of starting your master's degree. *

- ◯ Band 5
- ◯ Band 6
- ◯ Band 7
- ◯ Band 8a
- ◯ Band 8b
- ◯ Band 8c
- ◯ Band 8d
- ◯ Band 9
- ◯ Other

9. Please select the grade of your current role. *

- ◯ Band 5
- ◯ Band 6
- ◯ Band 7
- ◯ Band 8a
- ◯ Band 8b
- ◯ Band 8c
- ◯ Band 8d
- ◯ Band 9
- ◯ Other

10. Please select the sector in which you worked at the time of completing your master's degree. *

- ◯ Public sector health care - NHS
- ◯ Public sector local authority
- ◯ Voluntary sector
- ◯ Private sector healthcare
- ◯ Private sector local authority/care services
- ◯ Higher education institute
- ◯ Other

11. Please state, to the nearest whole year, how long you have been qualified in your profession. *

The value must be a number

12. Please state the clinical specialty in which you work. *

13. Please select the primary reason you undertook a master's degree. *

- Career progression - clinical knowledge & skill
- Career progression - leadership development
- Career progression - research and development
- Career progression - education/academia
- To improve pay-related income
- Personal interest
- Employer expectation
- Other

14. Please state the year in which you completed your master's degree (YYYY). *

The value must be a number

15. Please select the type of master's degree you completed. *

- Master of Art (MA)
- Master of Science (MSc)
- Master of Philosophy (MPhil)
- Master of Research (MRes)
- Master of Business Administration (MBA)
- Other

16. Please state the full title of your Master's degree research dissertation. *

17. Please select the statement which best describes how you formed the question for your master’s degree research dissertation. *

- ◯ I formed the question from my own ideas
- ◯ I formed the question based on published research priorities
- ◯ I formed the question through discussion with my supervisor
- ◯ I selected/was issued a pre-set question formed by the university department
- ◯ I selected/was issued a pre-set question formed by a collaboration of the university and a health-care organisation
- ◯ The research question and project was pre-set as part of a regional or national research collaborative
- ◯ Other

18. Please select the research method(s) approach(es) used to conduct your master's research dissertation. *

Select all the options that are applicable.

- [ ] Evidence synthesis (literature review, scoping review etc)
- [ ] Service evaluation
- [ ] Qualitative - Survey/Questionnaire
- [ ] Qualitative - Interview/focus group
- [ ] Action/participatory research
- [ ] Quantitative - secondary data analysis
- [ ] Quantitative - observational study
- [ ] Quantitative - experimental study
- [ ] ______

Other

19. Please select the options that describe how you promoted and shared the findings from your master’s degree research dissertation. *

Please select the 'in progress' option if this work has been submitted and is awaiting approval.

	Yes	No	In progress
Presented at local research symposium	◯	◯	◯
Conference poster	◯	◯	◯
Conference presentation	◯	◯	◯
Published abstract	◯	◯	◯
Published findings in clinical interest group journal/newsletter	◯	◯	◯
Published manuscript in peer reviewed journal	◯	◯	◯
Published a blog	◯	◯	◯
Published in a profession-specific circulation	◯	◯	◯

20. Please describe the impact your master’s degree research dissertation and the research-related course content has had on your professional development. *

	Stongly agree	Agree	Neutral	Disagree	Strongly disagree
Improved confidence	○	○	○	○	○
Improved clinical / leadership capability	○	○	○	○	○
Improved research capability	○	○	○	○	○
Influenced career progression (e.g. promotion)	○	○	○	○	○
Increased income	○	○	○	○	○
No impact	○	○	○	○	○
Deterred me from future engagement with research	○	○	○	○	○

21. Please use the space below to record further information about the impact your research dissertation and the research-related course content had on your professional development.

22. Please select the options that describe the impact your master's degree research dissertation and the research-related course content has had on the services in which you work. *

	Strongly agree	Agree	Neutral	Disagree	Strongly disagree	Not applicable
Improved service user outcomes /experience	○	○	○	○	○	○
Improved your own practice	○	○	○	○	○	○
Improved team practice	○	○	○	○	○	○
Developed/improved clinical pathway	○	○	○	○	○	○
Changed organisational policy or guideline	○	○	○	○	○	○
Influenced national guideline	○	○	○	○	○	○
No impact	○	○	○	○	○	○

23. Please use the space below to record further information about the impact your research dissertation and research-related course content had on the services you work in.

24. Please select the options that describe your engagement in research activity since completing your master's degree. *

	Yes	No
I embarked on a higher research degree (PhD)	○	○
I have taken part in formal research projects	○	○
I have received research grants/funding	○	○
I have published research papers	○	○
I have protected research time in my role	○	○
I work full-time in research/academia	○	○
I help others to develop research skills and undertake projects	○	○
I did not engage in research activity afterwards	○	○

25. Please use the space below to record further information about your research activity following completion of your master's degree.

26. Reflecting back on the completion your master's degree research dissertation project, please select the options that apply to you. *

	Strongly agree	Agree	Neutral	Disagree	Strongly disagree
My research question /topic addressed a real-world problem.	◯	◯	◯	◯	◯
I was expected to publish my findings in a research journal	◯	◯	◯	◯	◯
I was expected to present my findings at a conference	◯	◯	◯	◯	◯

27. Reflecting back on the expectations you had following completion of your master's degree, please select the options that apply to you. *

	Strongly agree	Agree	Neutral	Disagree	Strongly disagree
I expected to gain a promotion following completion of my master's degree	◯	◯	◯	◯	◯
I expected to undertake further research activity after completing my master's degree	◯	◯	◯	◯	◯
I expected support from my employer to continue research activity after competing my master's degree	◯	◯	◯	◯	◯

This content is neither created nor endorsed by Microsoft. The data you submit will be sent to the form owner.

Microsoft Forms
