# Supplementary material for: Evaluating the perceived impact and legacy of master’s degree level research in the allied health professions: a UK-wide cross-sectional survey
Source: BMC Med Educ. 2024 Jul 12;24:750. doi: 10.1186/s12909-024-05582-0 (PMC11241887; doi:10.1186/s12909-024-05582-0)
Supplement: Supplementary file 3 — Supplementary Material 3. [file 12909_2024_5582_MOESM3_ESM.docx]

**Additional file 3**

**Evaluating the perceived impact and legacy of master's degree level research in the allied health professions: A UK-wide cross-sectional survey.**

**Sample size calculation:**

Confidence level 95%

Margin of error 5%

Qualifying population 10% (Est. % UK AHP population master’s degree award)

Population size 120,000

Sample size 139
